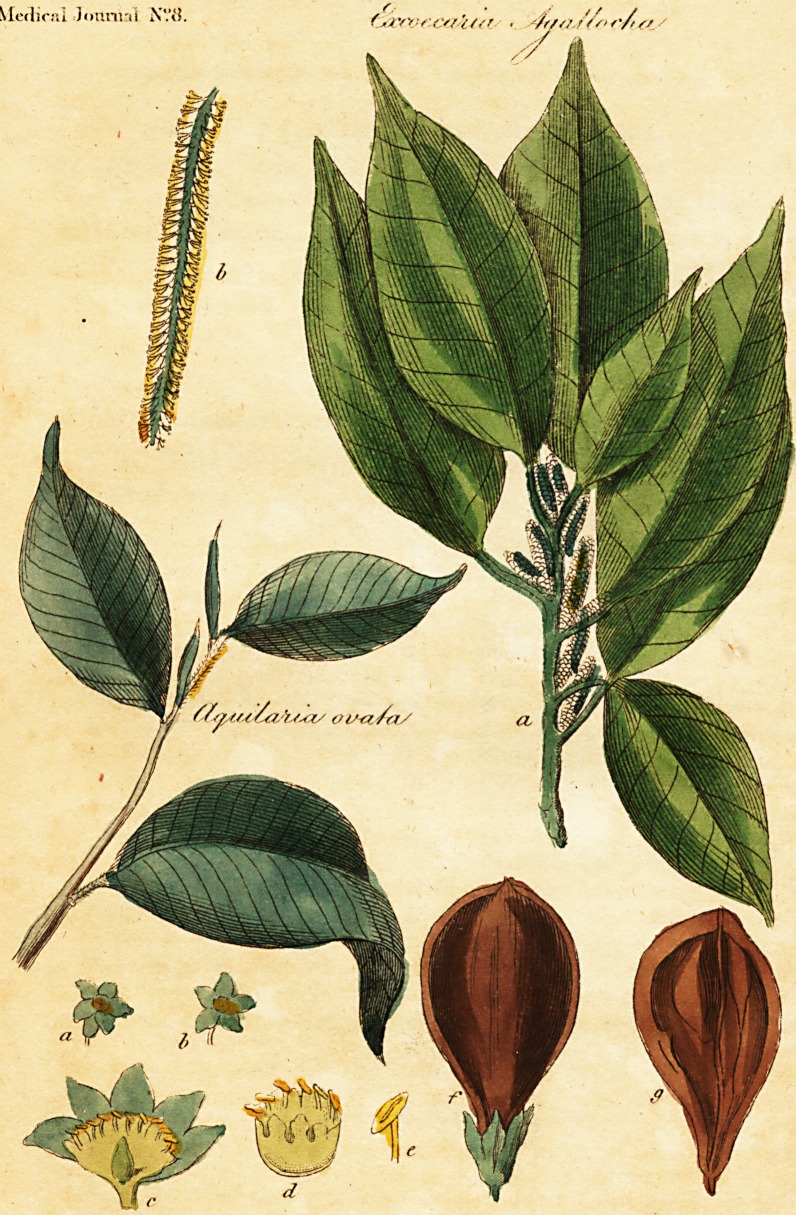# Medical and Physical Intelligence

**Published:** 1799-10

**Authors:** 


Printed for R. Phillips, 71 St. Paul's Church Yard.

### MEDICAL AND PHYSICAL INTELLIGENCE,

### (Original and Selected.)

### *On the aromatic Wood of Aloe, with a Botanical Description of the Excoecaria Agallocha, and the Aquilaria Ovata:— By Professor* WILDENOW, *of Berlin.*

[Illustrated by a coloured Plate.]

THE agallochum, which is known by the name of aloe-wood, or the aromatic aloe, is one of the most valuable spices imported from the East, and has been in high estimation, even in the earliest ages. According to the different species of the plant, it is called *lignum aloes, agallochi veri, aquilæ,* and *calambac,* all of which differ remarkably in their sensible properties. It is, however, very difficult to ascertain the botanical character of these various species.

The aromatic wood of aloe is properly a resin, which has pervaded the pores of the tree. The genuine sort of this resinous wood is as precious as gold, and is used only by the great and affluent in the East-Indies, China, and Japan, as an agreeable perfume, with which they fumigate houses: hence it is but rarely imported into Europe. It is black, and variegated with grey veins, swims on water, and if strongly rubbed on glass, leaves behind resinous particles, which neither water, saliva, expressed oils, nor an alkaline lye, but spirit of wine only, will dissolve and remove. Its odour is very grateful.

This substance has, in former times, been much used as a medicine, not only in biliary complaints, diseases of the liver and stomach, and dysentery, but likewise as a remedy for the maw-worm; but at present it is entirely neglected—a revolution to which several of the ancient remedies have been subject, and which is likely to take place with many of the modern.

Father DE LOUREIRO assures us, that he discovered, in the province of Conchin-China, the tree which affords the true aloe. This aromatic wood is found in resinous masses, only in old, half-decayed, hollow trees. According to this writer, the tree belongs, in the system of LINNÆUS, to the first division of the tenth class, the Decandria Monogynia: he called it *Aloexylum Agallochum.* It grows on the highest mountains of Conchin-China, on the banks of the river Lav, which flows through the whole of that province. Loureiro had no opportunity of seeing the blossoms on the tree; he could only once obtain them dried, and transmitted by a friend, so that the parts of fructification were much bruised and lacerated on a long journey, and he could with difficulty give the following description:—

ALOEXYLUM AGALLOCHUM.

Differ. spec. Aloe. *foliis* lanceolatis, alternis: *pedunculis* polyfloris, terminalibus.

Hab.

Hab. *Arbor* magna: *trunco* et *ramis* erectis, altiſſimis: *cortice* cannabino, fuſco, glabro, nec craſſo.

*Folia* lanceolata, octo polyces longa, integerrima, plana, glabra, ſub-coriacea, alterna, petiolata. *Flos* terminalis, pedunculis polyfloris.

Uſus. Ligni hujus ſuffimenta inter omnia maxime æſtimantur apud nationes Orientales. Ex arboris cortice fit vulgaris charta, in quâ Conchin-chinenſes ſcribunt, ſicut in Japonia fit ex cortice Mori.

Virtus medica. Excitans, corroborans, cephalica, cardiaca. Suffitus valet contra vertiginem & paralyſim. Pulvis cohibet vomitum et fluxus ventris, præcipuè Lientricos, quod non propriè aſtringendo, ſed corroborando agit.

This tree is not of a poiſonous nature, and yields no milky ſap when perforated. With reſpect to the *genuine* wood of aloe, Loureiro maintains that the various ſpecies differ remarkably, both in colour and flavour. By ſome botaniſts, this aromatic wood has been confounded with the *Lignum Aquilæ*, which is likewiſe eſteemed for its agreeable odour, and like the Agallochum verum, Calambac, and Garo de Malacca, affords different ſpecies of perfume. Kaempfer and Lamarck have given a particular deſcription of the genuine aromatic aloe. Cavanilles, an eminent botanical writer, calls the plant that produces this valuable drug,

AQUILARIA OVATA.

*Character genericus.*

Calyx turbinatus, coriaceus, ſemiquinquepartitus, laciniis ovato-acutis patulis perſiſtentibus.

Corolla nulla.

Stamina: urceolus calyci imo adhærens, monophyllus, quinquepartitus, laciniis craſſis tomentoſis profunde bifidis, adeo ut decemfidus appareat. Ex ſingulis diviſionibus totidem adſurgunt filamenta breviſſima ſquamulis breviora: antheræ decem, oblongæ verſatiles.

Germen in ſquamulorum centro et fundo calycis, ovatum, coronatum ſtigmate brevi ſimplici.

Fructus: capſula pyriformis, lignoſa, bivalvis, bilocularis: diſſepimentum bipartibile, innaſcens valvis medio ſeptiferis: ſuturam ambiente membranula brevi.

Semina ſolitaria nigra corpore ſpongioſo circumdata; alterum ſæpe abortivum.

Habitat in Malacæ montibus. *

*Differ. Spec.* A. Foliis alternis, ovatis, mucronatis.

Garo de Malaca. *Lamarck. Encycl. Tom. I. p.* 49. *Tom. II. p.* 610.

Arbor, cujus rami conſtant ligno albicante-luteo, cortice griſeo tecti: villoſique tenerrimis ſummitatibus.

Folia alterna, petiolis ſuſtentata brevibus piloſis, ovata, terminata mucrone, integra, glaberrima, uninervia, nervo ramoſo, veniſque ſubtiliſſimis.

Stipulæ nullæ.

Florum ſitus et numerus mihi ignotus.

Of this plant we have given our readers an accurate copy taken from the work here quoted: and it is remarkable, that Loureiro deſcribes probably the ſame plant in his "*Flora Conchinchinenſis*," under the name of *Ophiſpermum Chinenſe*; as it differs from the former only by a long filiform ſtyle, and a bipartite ſtigma. Perhaps this apparent difference ariſes from the

* Vid. *Cavanilles Dissertat.* VII. pag. 377. tab. 224.

the flower of the plant described by CAVANILLES, having been injured by being dried and compressed between paper. He has given no description of the calyx, and is of opinion, that it does not exist. Professor WILDENOW, however, supposes that the specimen in question is in this respect incomplete. The fruit is a compact, ligneous, oviform, compressed, two-celled capsule. Each cell contains a seed with a fungous edge. According to LINNÆUS, this plant also belongs to the first order of the tenth class, the Decandria Monogynia.

SONNERAT and KAEMPFER assure us, that the genuine wood of aloe, which is so highly valued, is obtained from this tree; and it is nevertheless probable, that several trees afford that precious drug; for all writers on the subject observe, that the difference among them, both in scent and colour, is remarkably great. For the same reason, Professor Wildenow is inclined to believe that the *Excoecaria Agallocha* of Linnæus, yields a similar drug, which has been introduced into commerce, under the specious name of aromatic aloe.—The reader will find a sprig of this tree, with male flowers, on the annexed plate; and as the work of Linnæus is generally known, we shall, instead of transcribing his description, translate that given by Professor Wildenow, in German.

"This tree (says he) grows wild in the East-Indies, and belongs to the third order of the twenty-second class of Linnæus, the Dioecia Triandria, that is, the male and female flowers grow on distinct stems, and the male flowers have three filaments. The trunk of this tree is of a very considerable size. The bark on the smaller branches is of a light brown colour, smooth, and somewhat cracked. The leaves come out alternate, are petiolate, ovate, sharp-pointed, entire, coriaceous, of a deep green colour, and glossy on the upper surface. The flowers are disposed axillary, in several spikelets. The male flowers are green, and, in their growing state, short and columnar. The filaments are gradually developed, become progressively longer, and have yellow anthers. Linnæus asserts, that the male catkins are composed of mere filaments, three of which uniformly stand together. But on the male flowers (which only the Professor had an opportunity of examining) he observed a roundish pointed scale, a small corolla of two petals, and three anthers.

"The female flowers are green, arranged in catkins, and formed like the male flowers. The germ is round, and has three styles. The fruit is a three-celled capsule. On cutting the tree, a quantity of milky sap flows from the orifice of the wound, and, if it be brought in contact with the eye, occasions blindness. In very old hollow stems, there is a resin which has penetrated through the brittle wood, and is likewise known in commerce, by the name of lignum aloes.

"From this account we may conclude, that the best and most valuable wood of aloe is obtained from the Aloexylum Agallochum; next to that, one of an inferior quality from the Aquilaria Ovata, and the most indifferent kind, from the Excoecaria Agallocha.

EXPLANATION OF THE PLATE.

*Excoecaria Agallocha:* *a.* A branch of the natural size, with the catkins just opened.

*b.* A catkin in full blossom.

*Aquilaria Ovata:* a foliated branch.

*a. b.* Two flowers of the natural size.

*c.* The flowers represented full blown, but somewhat magnified, so as to display the nectary, with the stamens, and the pedunculus.

*d.* The

*d.* The nectary magnified with the filaments.

*e.* A filament, with the anther much magnified.

*f.* The fruit, with the calyx of the natural ſize.

*g.* The ſame diſſected.

---

We are indebted to a Correſpondent whoſe paper is ſigned "PHILO," and is dated Auguſt 26, for the following communication, which, we preſume, will not be overlooked by our botanical readers:

The ſubject is the *Meſembryanthemum Pinnatifidum.* See "*Curtis's Botanical Magazine,*" pl. 67. If that excellent botaniſt had not publiſhed his account of this plant ſo ſoon, when he had only ſeen a very young ſpecimen of it, he would probably have ſuperſeded what I have to ſay; for whatever was curious ſeldom eſcaped the obſervation of his penetrating eye. I am not ſufficiently acquainted with the genus Meſembryanthemum, to know to what degree the different ſpecies vary with regard to the form of the ſeed-veſſels, but I believe the difference is very conſiderable, as in ſome ſpecies they are five-celled, in ſome four, and in ſome ten-celled, correſponding with the number of ſtyles; but having had one of this ſpecies ſtand in my window for ſome months this ſummer, I have had frequent opportunities of obſerving it. The whole habit of the plant, and even ſimilar cryſtalline points all over the ſtalks, beſpeak at firſt ſight, it's near relationſhip to Meſembryanthemum cryſtallinum, or common ice-plant. Like this too, it is an annual, contrary to the generality of the genus. The young botaniſt, however, as yet unacquainted with the habits of plants, and their natural families, might be much puzzled to find it in his ſyſtem, as it has for the moſt part only five, ſometimes ſix ſtamens*. This circumſtance ſeems to ſhew the natural affinity between the genus craſſula and this. The petals are far leſs numerous than in moſt of the genus, generally ſixteen.

Mr. Curtis has obſerved, that if the weather be fine, the bloſſoms open about two or three o'clock in the afternoon, moſt of the ſpecies open ſooner, but in general not till about noon, whence the name, which ſignifies noon or mid-day flower. My plant ſtood in a bow-window fronting the Eaſt, and had the morning ſun full upon it till twelve o'clock, and no longer, yet it never opened till two o'clock in the afternoon, at which time it was not expoſed to the rays of the ſun; in its time of flowering, therefore, it appears to be influenced by ſome other cauſe than either heat or light, as are many other plants: ſee the Horologium Floræ, in Linnæus's *Philoſophia Botanica.* But the circumſtance which attracted my attention moſt, and indeed is the cauſe of my troubling you with theſe remarks was, the ſeed-veſſel. This affords a good inſtance of what I underſtand by capſula turbinata. Its flat top, or umbilicus, is neatly marked with five rays, diverging from a point in the center; as the capſule ripens it becomes ſomewhat diſhed, ſo that it will hold a little water, and the foot-ſtalk is bent up to hold it in a horizontal poſition. While the weather continues fine, the fruit dries, but does not open; but when the rain falls, a little water lodges in the diſhed top, ſoaks in, and now the five triangular valves, the points of which before met in the center, fly open, expand horizontally, and are even bent backwards, bringing with them an internal tranſparent membrane, neatly jagged at the edges, the whole having the appearance of a full-blown flower, of which the outer valve forms the calyx, the inner membrane the corolla.

The

---

* It should be remarked, that this, and all the observations, were made upon one individual.

The cells containing the ſeeds are thus in part laid open, expoſing them to be waſhed out by the rain and diſperſed; I ſay in part, becauſe they are not entirely uncovered, part of the inner membrane remaining attached to the diviſions of the cells (diſſepimenta), forming a five-radiated ſtar, by which the ſeeds are prevented from being all ſuddenly waſhed away. When the rain ceaſes, and the capſule becomes dry, the valves cloſe as before, and may be made to open at pleaſure, by dropping a little water into the diſhed top of the capſule; as the water dries away, the valves cloſe again, and thus this ſemblance of a flower may be made to expand or ſhut up at pleaſure *. I made a little attempt to improve the ſpectacle by colouring the tranſparent membrane, to make it more nearly reſemble a corolla, but for want of proper materials, I ſucceeded very badly. Could the outer valves be ſtained green, and the inner membrane crimſon, yellow, or any other ſhewy colour, in ſuch a manner that the neceſſary wetting ſhould not make the colours run one into another, it would make an amuſing recreation.

---

Dr. Scherer, of Jena, in a letter to Van Mons, on the *chemical action of light*, obſerves that he inſerted in the ſeventh Number of his Journal, a Memoir of Count Rumford, in which that philoſopher expreſſes his doubts of light having the power to act chemically on bodies. Among the experiments he adduces to ſupport this opinion, the moſt remarkable is that in which charcoal has effected, in darkneſs, as complete a reduction of the ſolution of gold, as it would have undergone in the preſence of light.— "*Annales de Chimie*," No. 91.

---

Dr. Scherer alſo communinates an account of an apparatus, by means of which bleaching may be executed with the oxygenated muriatic acid alone, as well as by the addition of ſoda. He proves that the ſolution of indigo is leſs diſcoloured, in proportion as the acid is more ſaturated with this alkali. *Ibid.*

---

On the preparation of the *muriat of barytes*, the ſame chemiſt remarks that he found the operation conſiderably ſhortened by uſing, what he calls, the native carbonat of England, or the *witherite*. By this means, not only the difficulty of ſeparating the barytes from the ſulphat of this earth is avoided, but the ſalt is prepared at half the ordinary expence.—*Ibid.*

---

Mr. Smith has ſome time ſince communicated, in the European Magazine, a very curious hypotheſis reſpecting the production of ſulphur; while he maintains that it is collected in Dumfrieſhire, at Moffat, and Harrowgate, from a ſoil compoſed of the remains of plants and vegetable earth; and that, during the act of vegetation, by ſome unknown proceſs of nature, the ſulphuric acid is generated, which combines either directly with ſome of the vegetable ſubſtances, or with ſoda, in the ſame manner as the different animal acids are formed.

"It is probable (remarks Dr. Scherer) that during putrefaction, the oxygen of the ſulphuric acid is firſt volatilized by the carbon and hydrogen, as that effect takes place during combuſtion; with this difference, however, that in the latter operation the liberated ſulphur is re-oxygenated, while during the former it combines with the ammonia, and conſequently, when this combination diſunites, it alſo combines with the hydrogen gas.—*Ibid.*

---

M. Fries,

* If put into warm water, the expanſion will be performed quicker.

M. Fries, of Rofingen, is preparing for the prefs, an Effay on the *Stœchiometry* of Richter, and alfo a continuation of his inquiry refpecting the *application of mathematics to chemiftry*. Thefe refearches are of greater importance than may be at firft conceived; and the doctrine of affinities, in particular, will derive much advantage from them; for, in chemical action, the affinity of bodies depends more on their compofition and decompofition, than on their occult qualities. The fame idea has been formerly adopted by Kirwan, but this learned chemift did not know how to make an extenfive application of it, fo that he failed in a great number of very delicate experiments.—*Ibid.*

M. Juch has informed Dr. Scherer, that he diftinctly perceived the fmell of *nitric acid* exhaled by the percuffion of *fugar*. He imagines that the atmofpheric air, by becoming partly difoxygenated, yields fufficient portions of azote and oxygen, to form this acid.—*Ibid.*

M. Lentin, of Gœttingen, afferts that the *falling ftar* is a new animal fubftance prepared in the ftomach of fome animal, where it acquires its gelatinous confiftence. He found in feveral fpecimens of this fubftance the thighs and other parts of frogs: hence he concludes, that it may probably be the mufcular fibre of that animal. The whole appeared, at firft, to diffolve by diftillation, and to form an aqueous, colourlefs liquid; but, towards the end of the experiment, there appeared a little empyreumatic oil, and a fubftance refembling carbon remained in the retort. The ftrained liquor had a very difagreeable fmell: it imparted to the turnfol a red colour, and he believed that this effect was produced by the zoonic acid. M. Lentin remarks, as a fingular circumftance, that this fubftance may remain for feveral months, expofed to the combined action of moifture and heat, without changing to a putrid ftate.—*Ibid.*

M. Gaertner has lately communicated to Dr. Scherer fome interefting obfervations on the conftituent parts of *urine*, and on the luminous property of *touchwood*. Thefe remarks are contained in the Chemical Journal edited by Scherer, but which is not yet come to hand.—*Ibid.*

M. Von Crell has lately announced in the laft mentioned Journal, that *carbon* is the bafis of the *boracic acid*.—*Ibid.*

### *Domeftic Intelligence.*

*It having been fuggefted to us, that a concife Account of the different Hofpitals, Infirmaries, and other Medical Inftitutions in Great Britain, would be acceptable to many of our Readers, and alfo tend to diffufe the benefits of the Healing Art; in compliance with this fuggeftion, we requeft our Correfpondents to furnifh us with fuch accounts of thefe Eftablifhments, as may feem likely to anfwer ufeful Purpofes. We fubmit the following outline of the Particulars: an Account of the Origin or Foundation of the Inftitution; a concife Hiftory of its Progrefs to the prefent Time; a Defcription of its prefent State, with refpect to Direction, Medical Officers, number of Pupils, Patients, &c. annually admitted.*

Dr. Bradley will recommence his Courfe of Lectures on the Theory and Practice of Phyfic, at the lecture-room, No. 102, Leadenhall-ftreet, on Monday the 7th of October, at fix o'clock in the afternoon.

Dr.

Dr. Crichton, of the Weftminfter Hofpital, will commence his ufual autumnal courfe of Lectures on the Theory and Practice of Phyfic, Chemiftry, and Materia Medica, on Monday the 7th of October. Thefe lectures will hereafter be delivered at No. 15, Clifford Street, Bond-Street.

---

Dr. Dennison and Dr. Squire, Men-midwives to the Lying-in Charity for delivering poor women at their own habitations, will commence their Lectures on the Theory and Practice of Midwifery, and the Difeafes of Women and Children, in the firft week of October, in the following order: Dr. Dennifon at the London Hofpital, and Dr. Squire at No. 2, Little Cloifters, under the Gate-way, Weft-Smithfield. Thefe lectures will be continued through the year, and the day of beginning each courfe advertifed in the public papers.

Gentlemen attending thefe lectures will find confiderable advantages in real practical midwifery.

---

Dr. Batty, of the Britifh Lying-in Hofpital, Brownlow-Street, and Phyfician to the Infant Afylum, will begin a Courfe of Lectures on the Theory and Practice of Midwifery, and the Difeafes of women and children, on Monday, October 7, at his houfe in Great Marlborough-Street.

---

Mr. Cruikshank and Mr. Wilson, will begin the winter courfe of their anatomical Lectures on Tuefday, October 1, at two o'clock, at their Anatomical Theatre, in Great Windmill-Street.

---

Mr. Wilson will begin his Lectures on the Principles and Practice of Surgery, on Monday, October the 7th, at feven o'clock in the evening, as ufual.

---

Mr. John Pearson, Surgeon of the Lock-Hofpital, Afylum, and Public Difpenfary, will commence his ufual courfe of Autumnal Lectures, on the Principles and Practice of Surgery, on Monday, October 7, at feven o'clock in the evening, at his houfe in Golden-Square.—Gentlemen who attend thefe Lectures, may have the advantage of exemplifying the general doctrines they fhall hear delivered, by attending the Chirurgical Practice at the Difpenfary.

---

Of the late Profeffor Gren's "*Elements of Chemiftry,*" in two volumes, which we have already noticed in our fifth Number, p. 514, we can now confidently promife a faithful and claffical tranflation, from the joint efforts of two learned chemifts, a German, and a native of this country. We have feen a fpecimen of the firft fheets of this excellent Compendium, from the Englifh prefs, and were informed that the work fhall appear towards the end of the prefent year; the plates are engraving by that eminent artift, Mr. Lowrie.

---

Dr. Willich and the Rev. P. Will propofe fpeedily to publifh a monthly work, entitled: *The Domeftic Magazine and Review*—on a plan entirely new, and to be embellifhed with plates. Particulars are ftated in a Profpectus, circulated by all bookfellers of refpectability in the three kingdoms.

## Figures and Tables

**Figure f1:**